# Co-Deregulated miRNA Signatures in Childhood Central Nervous System Tumors: In Search for Common Tumor miRNA-Related Mechanics

**DOI:** 10.3390/cancers13123028

**Published:** 2021-06-17

**Authors:** George I. Lambrou, Apostolos Zaravinos, Maria Braoudaki

**Affiliations:** 1 Choremeio Research Laboratory, First Department of Pediatrics, National and Kapodistrian University of Athens, Thivon & Levadeias 8, Goudi, 11527 Athens, Greece; glamprou@med.uoa.gr; 2 Department of Life Sciences, European University Cyprus, Diogenis Str., 6, Nicosia 2404, Cyprus; 3 Cancer Genetics, Genomics and Systems Biology Group, Basic and Translational Cancer Research Center (BTCRC), Nicosia 1516, Cyprus; 4 Department of Clinical, Pharmaceutical and Biological Science, School of Life and Medical Sciences, University of Hertfordshire, College Lane, Hatfield AL10 9AB, Hertfordshire, UK

**Keywords:** central nervous system, CNS tumors, pediatric CNS tumors, childhood CNS tumors, miRNA, common mechanics, microarrays

## Abstract

**Simple Summary:**

Childhood tumors of the central nervous system (CNS) constitute a grave disease and their diagnosis is difficult to be handled. To gain better knowledge of the tumor’s biology, it is essential to understand the underlying mechanisms of the disease. MicroRNAs (miRNAs) are small noncoding RNAs that are dysregulated in many types of CNS tumors and regulate their occurrence and development through specific signal pathways. However, different types of CNS tumors’ area are characterized by different deregulated miRNAs. Here, we hypothesized that CNS tumors could have commonly deregulated miRNAs, i.e., miRNAs that are simultaneously either upregulated or downregulated in all tumor types compared to the normal brain tissue, irrespectively of the tumor sub-type and/or diagnosis. The only criterion is that they are present in brain tumors. This approach could lead us to the discovery of miRNAs that could be used as pan-CNS tumoral therapeutic targets, if successful.

**Abstract:**

Despite extensive experimentation on pediatric tumors of the central nervous system (CNS), related to both prognosis, diagnosis and treatment, the understanding of pathogenesis and etiology of the disease remains scarce. MicroRNAs are known to be involved in CNS tumor oncogenesis. We hypothesized that CNS tumors possess commonly deregulated miRNAs across different CNS tumor types. Aim: The current study aims to reveal the co-deregulated miRNAs across different types of pediatric CNS tumors. Materials: A total of 439 CNS tumor samples were collected from both in-house microarray experiments as well as data available in public databases. Diagnoses included medulloblastoma, astrocytoma, ependydoma, cortical dysplasia, glioblastoma, ATRT, germinoma, teratoma, yoc sac tumors, ocular tumors and retinoblastoma. Results: We found miRNAs that were globally up- or down-regulated in the majority of the CNS tumor samples. MiR-376B and miR-372 were co-upregulated, whereas miR-149, miR-214, miR-574, miR-595 and miR-765 among others, were co-downregulated across all CNS tumors. Receiver-operator curve analysis showed that miR-149, miR-214, miR-574, miR-595 and miR765 could distinguish between CNS tumors and normal brain tissue. Conclusions: Our approach could prove significant in the search for global miRNA targets for tumor diagnosis and therapy. To the best of our knowledge, there are no previous reports concerning the present approach.

## 1. Introduction

A central nervous system (CNS) tumor begins when healthy cells in the brain or the spinal cord change and grow out of control, forming an either benign or cancerous tumor. The movement and cognition of patients suffering from a CNS tumor are affected, making it challenging to treat, because the tissues around the tumor are often vital to the body’s functioning [1]. About 1200–1500 new cases of CNS tumors are diagnosed per year in the US alone, with an equal ratio between the two sexes. More than 90% of primary CNS tumors occur in the intracranial cavity affecting the brain, meninges, epiphysis, optic nerve, and parapharyngeal areas.

Childhood CNS tumors differ greatly from other childhood neoplasms, as CNS tumors are related to high mortality rate if the tumor is unable to be removed surgically. The treatment of CNS tumors in infants and young children may be especially challenging because a child’s brain is still developing. However, more than half of these patients are disease-free five years after their diagnosis. With this increase in survival, these children are expected to have serious, permanent, neurological, cognitive, endocrinological and psychological problems after extensive radiotherapy treatment. Therefore, the life quality of long-term survivors needs to be improved. An interdisciplinary approach in managing brain tumors in children is important to improve treatment, especially in benign tumors, and in low-grade children malignancies.

MicroRNAs (miRNAs) are endogenous single-stranded RNA molecules ranging from 20 to 25 nucleotides in size, and deriving from larger precursors by maturation [2]. They play an important role in modifying the sequence, structure and expression of messenger (m)RNAs and at the same time, they affect protein translation. MiRNAs control cell proliferation, differentiation, apoptosis, angiogenesis, epithelial-to-mesenchymal transition (EMT), metastasis and metabolic pathways in cancer, including CNS tumors [3]. Thus, through their expression profiles, they can be used as biomarkers for the prognosis, diagnosis and treatment of different types of cancers [2].

In different cancers, miRNA expression is deregulated by a variety of mechanisms, including amplification, deletion, mutations, epigenetic silencing [4,5,6], or loss of the expression of their transcription factor [7,8]. Several miRNAs are distributed in fragile or cancer-related genomic regions [9]. In addition, they have been shown to function either as oncogenes or tumor suppressor genes [10,11]. MiRNAs can simultaneously target oncogenes and tumor suppressor genes in cancer cells. Therefore, the regulation of several signaling pathways is cooperatively being carried out by a combination of different miRNAs. Their oncogenic or tumor suppressor activity depends heavily on their cellular environment. For example, the miR-29 family exhibits tumor suppressor activity in lung cancer via targeting the DNA methyltransferase DNMT3A, while it has oncogenic activity in breast cancer, by targeting the DNA methyltransferases DNMT3B and/or ZFP36 [12].

Τhe identification of free circulating miRNAs from the whole blood, plasma or serum, can be successfully used in cancer research, and despite the heterogeneity in circulating tumor cells (CTCs) [13], miRNA profiling can be used as a prognostic and therapy-monitoring tool in childhood CNS tumors [14,15]. MiRNA profiling has been used to detect changes in neurons [16] and in some cases of pediatric CNS tumors they have also been used as diagnostic and therapeutic molecules [17,18]. Nevertheless, there is still a lot more to learn regarding their role in CNS tumors. New insights on the role of miRNAs in CNS tumors could help us understand better their pathology, etiology and treatment.

In this study, we hypothesized that different childhood tumors of the CNS could share similar miRNA expression patterns. We explored various GEO datasets containing expression data from different types of pediatric brain tumors, and we identified miRNAs that could serve as candidate biomarkers in their diagnosis, prognosis and therapy.

## 2. Materials and Methods

### 2.1. Collection and Analysis of Variables

Along with the microarray data we collected categorical variables for all tumor and control samples. In particular, we collected the following categorical variables: gender, age, country of origin of the study, survival at biopsy, diagnosis, anatomic location in the brain, tumor grade, developmental status (i.e., based on the subject’s age), sampling (if the biopsy took place at diagnosis or relapse), and clinical outcome.

### 2.2. Microarray Samples

We mined microarray data from the publicly available databases Gene Expression Omnibus (https://www.ncbi.nlm.nih.gov/geo/, accessed on 5 September 2020) (GEO) and ArrayExpress (https://www.ebi.ac.uk/arrayexpress/, accessed on 5 September 2020) which contain experiments on childhood CNS tumors. To this end, we combined the keywords: “childhood”, “CNS”, “brain tumor”, “primary”, “embryonal”, “central nervous system tumor”, [(pediatric tumor miRNA) AND “Homo sapiens”[porgn:__txid9606]], [(pediatric medulloblastoma miRNA) AND “Homo sapiens”[porgn:__txid9606]], [(pediatric astrocytoma miRNA) AND “Homo sapiens”[porgn:__txid9606]], [(pediatric ependymoma miRNA) AND “Homo sapiens”[porgn:__txid9606]]. From the recovered experiments (GEO Series) we selected only those concerning miRNA expression. Our search finally included 10 different series, as well as in-house microarray experiments concerning miRNA expression. In total, we collected data from 451 CNS tumor samples for further analysis, yet 12 samples were removed because they included patients older than 60 years old (Table 1).

### 2.3. Microarray Data Pre-Processing

**Figure 1:**
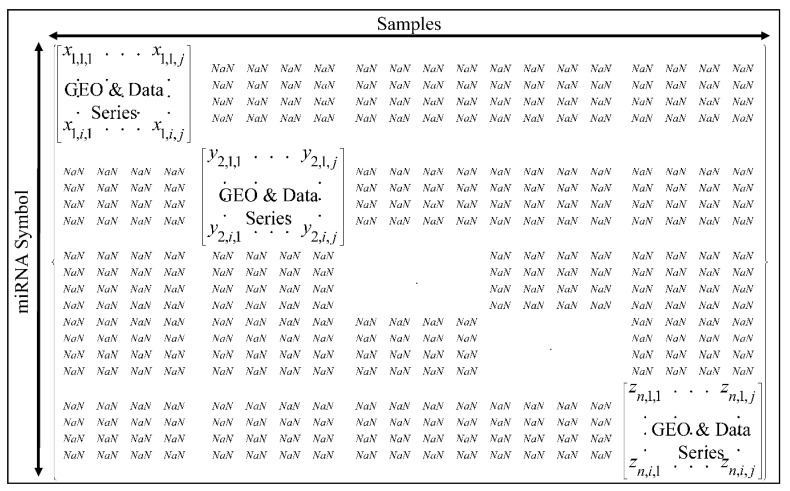
The hyper-matrix containing all data series for further processing, where *n* is the data series, *i* is the miRNA and *j* the respective sample.

The extracted microarray data were entered into a Microsoft Excel^®^ file. In order to identify the common miRNAs across all series we used the miRNA symbols as the common “denominator” across all series. Since each series had multiple occurrences of miRNAs, they were entered in a diagonal form in a hyper-matrix (Figure 1), in which the rows correspond to miRNA symbols, columns correspond to samples and the individual series were inserted diagonally. Empty cells were replaced with “NaN”.

### 2.4. Microarray Data Post-Processing

Microarray data were processed in Matlab^®^. They were initially background corrected using the multiplicative background correction (MBC) approach [26]. Specifically, MBC subtracts the logarithmic estimates of the background intensity from the logarithmic foreground intensity. Where no background data were provided in the GEO data series, microarray data were considered as background corrected and further correction was applied. In particular, the data series for which no background correction was applied were: GSE34016, GSE62367, GSE63319, GSE66968, and GSE84747.

After background correction, negative values were removed and replaced with “NaN” values. Our intention was to find miRNA expression, even those of low expression values. It is possible that not only those values that are of great difference are of importance, but also that those that have very low expression values could be of biological importance.

Microarray data normalization was then performed using three algorithms: (a) Loess [27], (b) Rank Invariant, and (c) Quantile algorithm. To account for batch effects, we divided the matrix elements by the global mean. To account for differences across series we used the log_2_-transformed ratio, which is performed as:

(1) $$\begin{array}{l} R_{i.j}=\log_{2}\left(\frac{x_{i,j}}{{\bar{x}}_{total}}\right)\\ X_{i,j}=2^{R_{i.j}} \end{array}$$

where, *R_i,j_* is the global mean-transformed ratio, *x_i,j_* is the expression value of gene *i* and sample *j*, *X_i,j_* is the restructured value of the *i*th gene and *j*th sample.

The three algorithms were compared for their efficiencies. In general, the Quantile algorithm performed better as compared to the other two. The normalized data are provided as supplementary data (Table S1).

To reduce the complexity of the data set, we followed the replicate averaging approach proposed by Uzman et al. [28]. We used the Student’s *t*-test [29] to identify the differentially expressed miRNA genes (DE miRNAs) across all tumor samples as compared to all control samples. The false discovery rate (FDR) was calculated as previously described [30,31,32]. The DE miRNA genes per experiment were identified at a confidence level of 95%. DE miRNAs were treated in two different ways. Data were further processed and analyzed as “ratios”, i.e., as gene expression values calculated as the log_2_-transformed ratio of each tumor sample over the mean of all control samples, using the following formula:

(2) $$E=\log_{2}\left(\frac{F_{Tumor,i,j}}{{\bar{F}}_{Controls,j}}\right)$$

where *E* is the expression value, *F_Tumor,i,j_* is the expression value of tumor sample *i* and miRNA *j*, ${\bar{F}}_{Controls,j}$ is the mean expression value of all controls and miRNA *j*.

In addition, data were also analyzed as “naturals”, meaning DEGs that are non-log_2_-transformed and thus including the control and tumor samples separately.

### 2.5. Unsupervised Classification Methods

DEGs were further analyzed for common expression patterns using classification methods, using Matlab^®^. To gain further insight into the gene expression data, we used unsupervised hierarchical clustering (HCL) and k-means classification [33,34]. HCL with dendrogram was used and correlations were calculated with Euclidean distance. K-means classification [33,34] was recently reported as one of the best performing clustering approaches for microarray class discovery studies [35]. We applied the squared Euclidean as a distance measure, since it is generally considered to be a more appropriate measure for use with k-means and found to outperform for ratio-based measurements [36]. We used 100 iterations and the optimal cluster number for the k-means algorithm was estimated using the Calinski–Harabasz criterion. Complete k-means clusters, centroids and sorted centroids [37] were utilized. The DE miRNAs were also classified based on their diagnosis categorical variable. In particular, the mean values of all samples with respect to the diagnosis was estimated and the resulting descriptive statistical measure was utilized for further k-means classification. Gene expression was also analyzed with respect to the chromosomal distribution of the DE miRNAs. We explored the mean expression per chromosome and heat-maps of chromosomal-related expression.

### 2.6. Common Expression Patterns in DE miRNAs

DE miRNAs were examined for possible common expression patterns, i.e., miRNAs that were either down- or up-regulated in all CNS tumor samples, irrespectively of the tumor diagnosis. The clusters revealed by unsupervised classification were examined separately. Each miRNA was counted for its occurrences for up- or down-regulation in all samples and the result was divided by the total number of samples, giving the percentage of up- or down-regulated samples of the respective miRNA. We have looked for miRNAs that were either up or down-regulated in all samples (100%), in 90–99% of all samples, 80–89% of all samples and 75–80% of all samples.

### 2.7. Receiver Operating Characteristic (ROC) Analysis

ROC curves and naïve Bayes classification were used to investigate the diagnostic ability of the co-deregulated miRNAs between CNS tumors and control samples. In the case of naïve Bayes classification, the algorithm used Bayes theorem, and (naively) assumes that the predictors are conditionally independent, given a class. Naïve Bayes classifiers assign observations to the most probable class (in other words, the maximum a posteriori decision rule).

### 2.8. Statistical Analysis

For comparisons between groups, the Student’s *t*-test and one-way analysis of variance (ANOVA) were performed for the continuous variables and chi-square tests were used for the categorical variables. Post hoc comparisons (adjusted with Bonferroni criterion) were also performed when significant differences (*p* < 0.05) of the variables in ANOVA tests were identified. A value of *p* < 0.05 (two-tailed) was set as the level of significance.

Chi-square test of independence was used to evaluate the association between patients’ characteristics. The characteristics that were found statistically significant were entered into a logistic regression model in order to evaluate the probability of having multiple positive reactions. The modeling of a quantitative variable based on one or more qualitative and quantitative parameters, was performed through linear regression. Multiple logistic regression was performed in order to evaluate the probability of having multiple positive reactions. The relative risk (RR), odds ratio (OR), and absolute risk (AR) were calculated.

### 2.9. Gene Ontology (GO) Enrichment Analysis

We performed GO enrichment analysis using the gprofiler [38] and WebGestalt web-tools [39]. Relations of the differentially expressed genes and the transcription factor binding motifs were further investigated using the Pubgene Ontology Database (www.pubgene.org, accessed on 5 September 2020). Gene definitions and functions were based on the National Institute of Health databases (http://www.ncbi.nlm.nih.gov/sites/entrez/, accessed on 5 September 2020).

### 2.10. Pathway Analysis

Pathway analysis was performed using the gprofiler [38] and WebGestalt web-tools [39].

## 3. Results

### 3.1. CNS Sample Cohort

In total, we analyzed 439 CNS samples (97 females and 165 males and 81 fetuses of unknown gender, as well as 171 samples for which no data were available). The total number of neoplasms were 195, irrespectively of the tumor type, and the control samples were 244. The majority of tumor samples were malignant (*n* = 154), followed by benign tumors (*n* = 41), mainly of grade I. Our sample cohort consisted of 53 grade I tumors, 18 grade II, 12 grade III and 92 grade IV tumors. Descriptive statistics of our sample cohort are summarized in Table 2.

### 3.2. Deregulated (DE) miRNAs

We identified 406 co-deregulated (DE) miRNA genes across all CNS tumor samples (*p*-value < 0.05 and FDR < 0.0019). DE miRNAs, were further processed to identify for similar patterns of expression and Gene Ontology (GO) enrichment analysis.

### 3.3. Chromosomal Distribution of DE miRNAs

**Figure 2:**
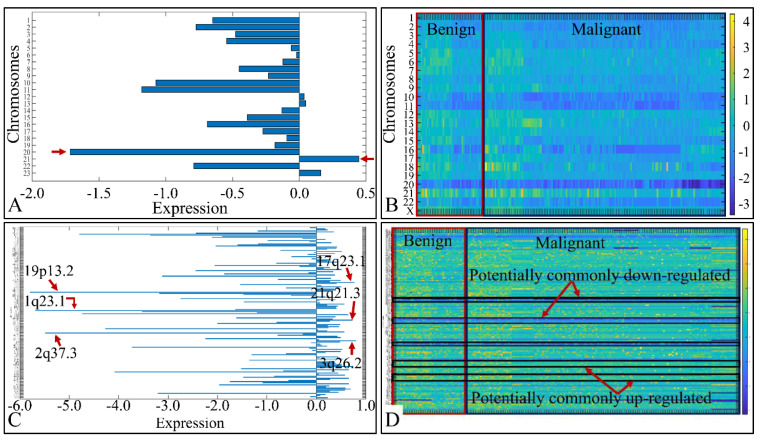
miRNA expression with respect to their chromosomal distribution (**A**) and location (**C**). The heat-maps depict the chromosome-related expression of the DE miRNAs (**B**), and their chromosomal location (**D**) (Legend: in sub-figure (**A**) the *x*-axis corresponds to miRNA expression and the *y*-axis to chromosome number. In sub-figure (**B**) the *x*-axis corresponds to tumor samples, the *y*-axis to chromosome number. In sub-figure (**C**)**,** the *x*-axis corresponds to miRNA expression and the *y*-axis to chromosomal locations. In sub-figure (**D**) the *x*-axis corresponds to tumor samples, the *y*-axis to chromosomal locations and the *z*-axis (the heat-map) to miRNA expression).

We analyzed the chromosomal distribution of the co-deregulated miRNAs across all CNS tumor samples. This included the estimation of the mean expression of the co-DE miRNAs per chromosome, as well as per chromosomal location (Figure 2). Although one would expect to find higher values of DE miRNA expression in longer chromosomes, such as chromosomes 1–5, we noticed that the highest expression was manifested by miRNAs located in chromosome 21. Also, chromosome 20 manifested the lowest miRNA expression in all CNS tumor samples, as expected due to its short chromosomal length (Figure 2A). In order to obtain a panoramic view of the chromosome-related expression across all samples, we created a heat-map with respect to chromosomal expression, which could provide a hint of the co-DE miRNAs (Figure 2B). In addition, when examining the mean miRNA expression with respect to their chromosomal location, we found that the co-upregulated miRNAs harbored primarily in chromosomal regions 17q23.1, 21q21.3 and 3q26.2. On the other hand, co-down-regulated miRNAs were primarily located in chromosomal regions 19p13.2, 1q23.1 and 2q37.3 (Figure 2C). We also created a heat-map for all chromosomal locations, which confirmed the possible presence of co-DE miRNAs (Figure 2D).

### 3.4. Unsupervised K-Means Classification

**Figure 3:**
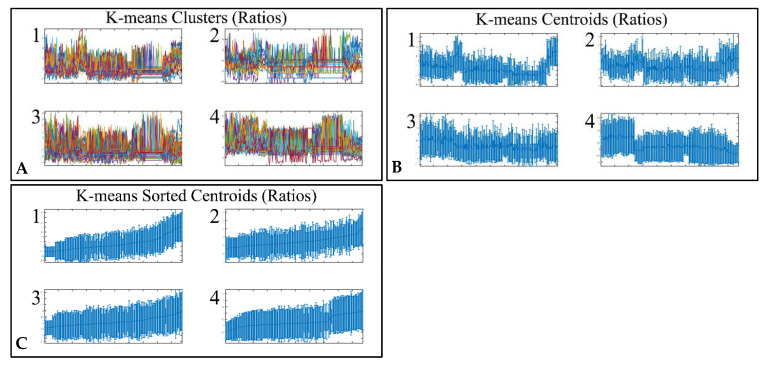
Unsupervised k-means clustering of DE miRNAs. Clustering resulted in four clusters (**A**), which are presented with their centroids (**B**) and the respective sorted centroids (**C**).

**Figure 4:**
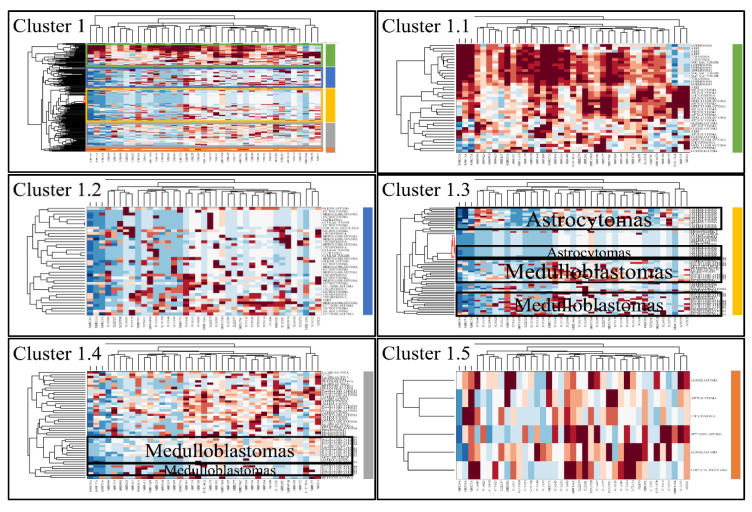
Hierarchical clustering (HCL) of cluster 1 as revealed previously by k-means clustering. The complete HCL is presented (Cluster 1) with its respective sub-clusters, denoted as clusters 1.1, 1.2, 1.3, 1.4 and 1.5. In sub-clusters 1.3 and 1.4, the DE miRNAs grouped together astrocytoma and medulloblastomas (at the right side of HCL for cluster 1, selected cluster are marked with different colors. Each sub-cluster is presented with its respective color).

**Figure 5:**
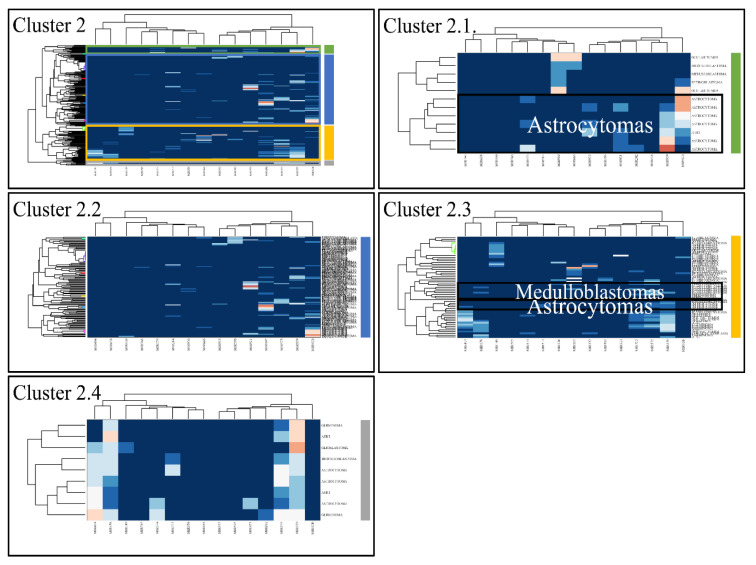
Hierarchical clustering (HCL) of cluster 2 as revealed previously by k-means clustering. The complete HCL is presented (Cluster 2) with its respective sub-clusters, denoted as clusters 2.1, 2.2, 2.3 and 2.4. In sub-cluster 2.3 miRNAs grouped together astrocytomas and medulloblastomas.

**Figure 6:**
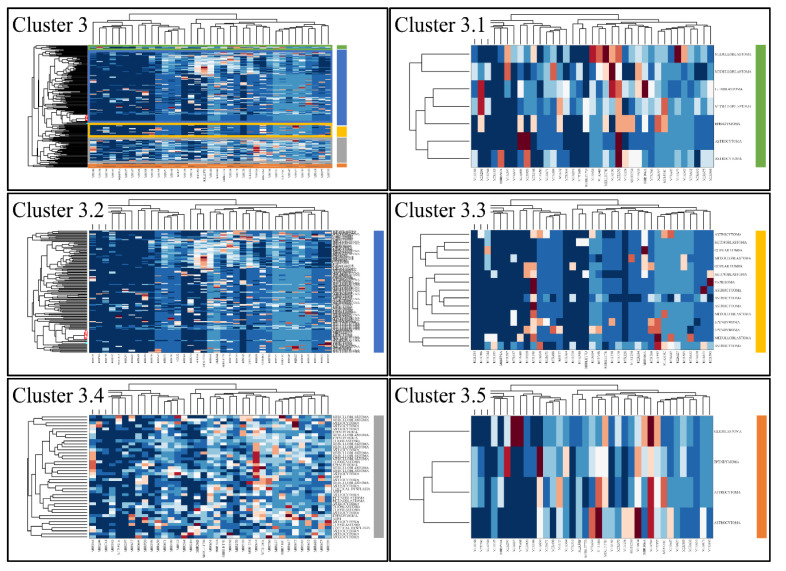
Hierarchical clustering (HCL) of cluster 3 as revealed previously by k-means clustering. The complete HCL is presented (Cluster 3) with its respective sub-clusters, denoted as clusters 3.1, 3.2, 3.3 3.4 and 3.5.

**Figure 7:**
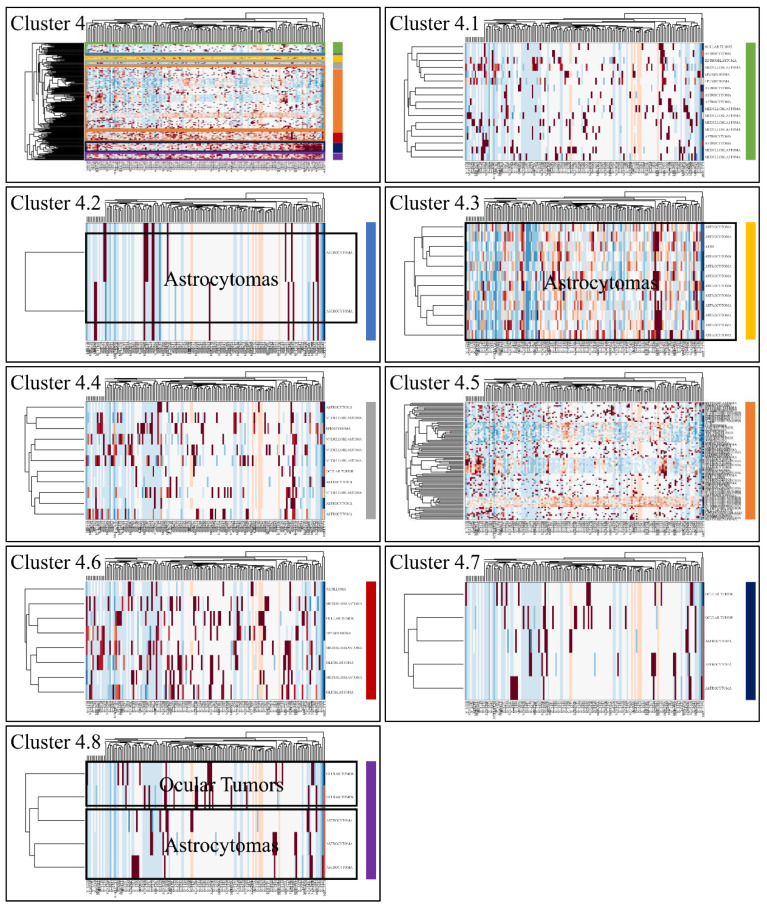
Hierarchical clustering (HCL) of cluster 4 as revealed previously by k-means clustering. The complete HCL is presented (Cluster 4) with its respective sub-clusters, denoted as clusters 4.1, 4.2, 4.3, 4.4, 4.5, 4.6, 4.7 and 4.8. In sub-cluster 4.2, 4.3 and 4.8 miRNAs grouped together astrocytomas and ocular tumors.

Classification algorithms are a valuable tool for the detection of common patterns across a gene expression dataset. Using k-means classification we clustered the DE miRNAs into four clusters, with no obvious pattern, corroborating the heterogeneity of the various types of CNS tumors (Figure 3). Each of them was then clustered hierarchically, to find potential patterns of expression (Figure 4, Figure 5, Figure 6 and Figure 7). As expected, the DE miRNAs did not successfully cluster the different types of CNS tumors. However, clusters 1.3, 1.4 (Figure 4), 2.3 (Figure 5), 4.2, 4.3 and 4.8 (Figure 7) manifested similar expression patterns between astrocytomas and medulloblastomas, suggesting common regulatory mechanisms between these two CNS tumor types.

### 3.5. Common DE miRNAs in Different CNS Tumor Types

We then examined each k-means cluster separately aiming to find co-deregulatory patterns of expression among all CNS tumors. We found several miRNAs that were globally up- or down-regulated in all tumor samples.

**Figure 8:**
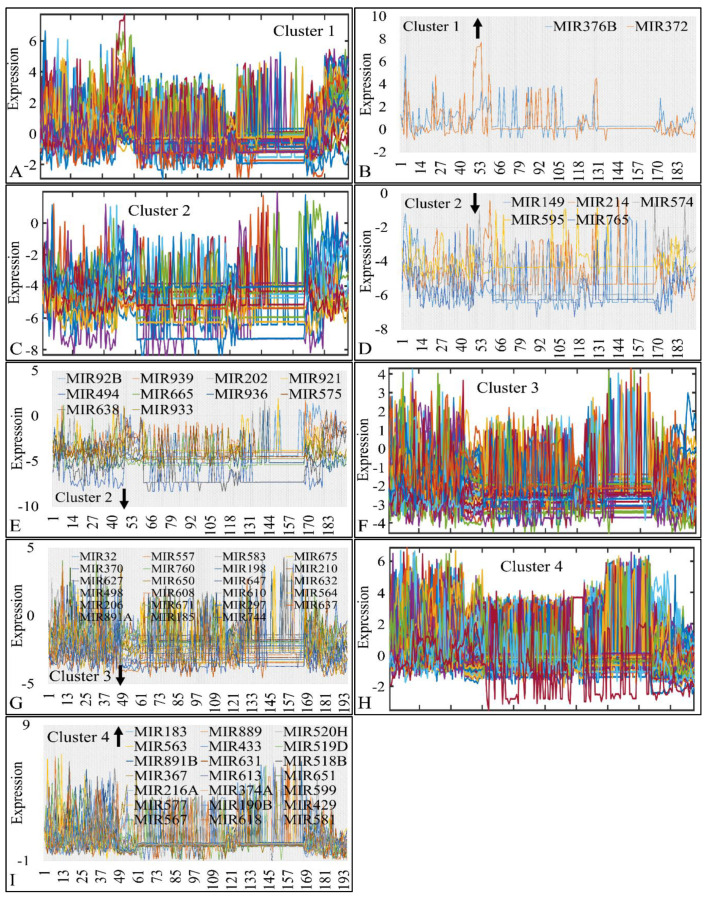
Common DE miRNAs across all CNS tumor samples. The co-DE miRNAs are presented in each k-means cluster (1–4). Each k-means cluster is presented separately along with the individual genes manifesting global up- or down-regulation. In particular, cluster 1 (**A**), manifested two globally up-regulated miRNAs (>75% and <100%) (**B**), cluster 2 (**C**), manifested five globally down-regulated miRNAs, which also consisted of a special case as they were down-regulated in all samples (100%) (**D**) and 10 globally down-regulated miRNAs in >90% of all samples (**E**), cluster 3 (**F**) manifested 23 globally down-regulated miRNAs (>90%) (**G**) and cluster 4 (**H**) manifested 21 globally up-regulated miRNAs (>90%) in all samples (**I**) (the arrow next to cluster number signifies the tendency of miRNAs in the respective cluster).

In particular, in cluster 1, MIR376B and MIR372 were globally up-regulated across most tumors (>75% and <100%) (Figure 8A,B). In cluster 2, MIR149, MIR214, MIR574, MIR595 and MIR765 were globally down-regulated across all CNS tumor samples (Figure 8C,D); ten miRNA genes were also found globally down-regulated in >90% of all samples (Figure 8E). In cluster 3 (Figure 8F) we detected 23 globally down-regulated miRNAs (>90%) (Figure 8G) and in cluster 4 (Figure 8H), 21 globally up-regulated miRNAs (>90%) (Figure 8I). The results of this analysis are also summarized in Table 3.

### 3.6. Descriptive K-Means

We analyzed the gene expression patterns, aiming to discover tumor groups according to their mean expression values. For example, we searched for an ascending or descending order of expression, based on the tumor’s characteristics, including tumor aggressiveness, diagnosis etc.

**Figure 9:**
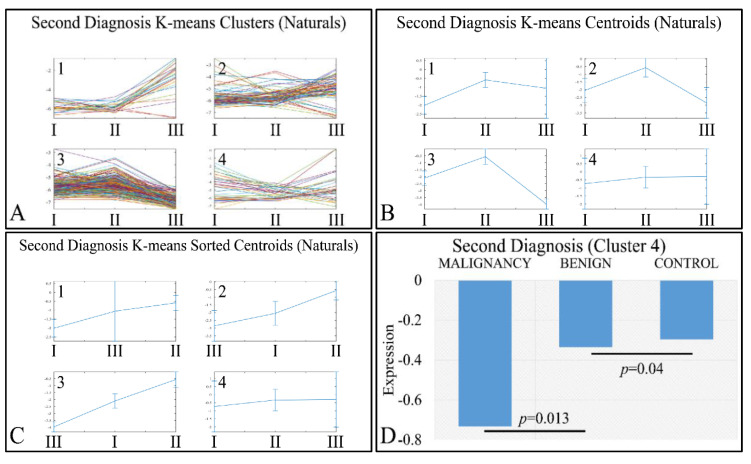
K-means clustering of DE miRNAs with respect to second diagnosis (controls, benign or malignant tumors), presented with the respective clusters (**A**), the centroids (**B**) and the sorted centroids (**C**). Cluster 4 manifested an ascending pattern from malignant tumors to controls (**D**). The expression levels were found to be significantly different between malignant and benign tumors, as well as between benign tumors and controls (Legend: I: Malignancy, II: Benign, III: Controls. Expression values range in the negative domain because we have used the log_2_-transformed natural values, in order to better visualize the differences).

Interestingly, a set of genes manifested an ascending order with respect to the classification of tumors as malignant, benign and controls (denoted as “Second Diagnosis”). Clustering analysis (Figure 9A–C) manifested four clusters, where the fourth cluster manifested the aforementioned ascending behavior (Figure 9D). The DE miRNAs in cluster 4 included: MIR1202, MIR1207, MIR1243, MIR1246, MIR1307, MIR1469, MIR1915, MIR2861, MIR3130, MIR3143, MIR3178, MIR3191, MIR3196, MIR3202, MIR320A, MIR320E, MIR3613, MIR3621, MIR3665, MIR3667, MIR3679, MIR3684, MIR4261, MIR4267, MIR4280, MIR4281, MIR4330, MIR494, MIR500B, MIR514B, MIR550, MIR560, MIR638 and MIRLET7A2.

**Figure 10:**
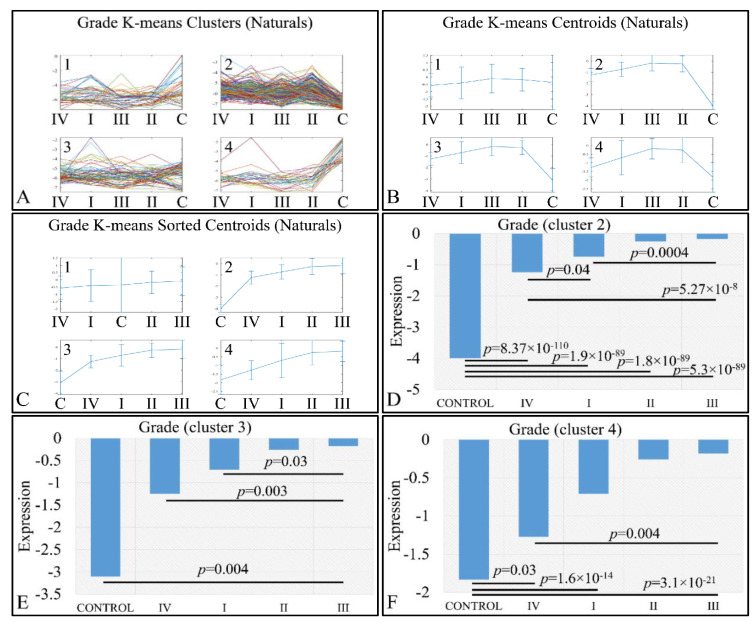
Expression patterns of miRNAs with respect to tumor grade, utilizing k-means clustering. Tumor grade includes the classification of tumors in five general categories: control and tumor grading from I to IV, which is presented with the respective clusters (**A**), the centroids (**B**) and the sorted centroids (**C**). Clusters 2 (**D**), 3 (**E**) and 4 (**F**) manifested an ascending motif from controls to grade IV, I, II and III. Significant differences between tumor grades are noted in the respective clusters (Legend: I: tumor grade I, II: tumor grade II, III: tumor grade III, IV: tumor grade IV).

An interesting pattern was also manifested with respect to tumor grading. We performed k-means clustering (Figure 10A–C), where clusters 2 (Figure 10D), 3 (Figure 10E) and 4 (Figure 10F) manifested as ascending pattern of expression. In particular, the control samples had the lowest expression levels, compared to the tumor samples, followed by tumors of grade IV and thereafter, by tumors of grades I, II and III. It apprears that the transition from the control samples to the most aggressive tumor types is direct, while the transition from lower tumor grades (I to III) follows a gradual pattern.

**Figure 11:**
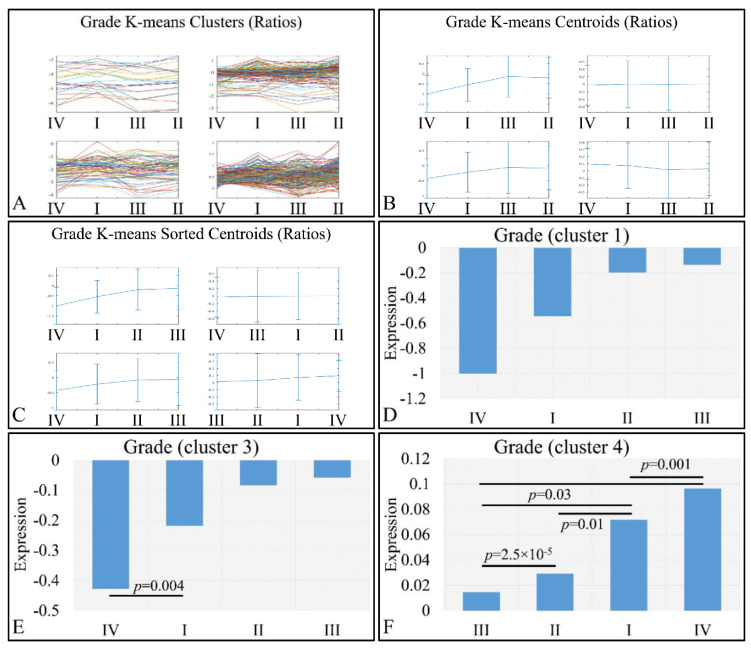
Expression patterns of miRNAs with respect to tumor grade utilizing k-means clustering and the log_2_-transformed ratios (tumors/controls). Tumor grade includes the classification of tumors into four general categories: tumor grading from I to IV, which is presented with the respective clusters (**A**), the centroids (**B**) and the sorted centroids (**C**). Clusters 1 (**D**) and 3 (**E**) manifested an ascending pattern from controls to grade IV, I, II and III, while cluster 4 manifested an ascending order from grade III to grade I and IV (**F**). Significant differences between tumor grades are noted in the respective clusters (Legend: I: tumor grade I, II: tumor grade II, III: tumor grade III, IV: tumor grade IV).

We also examined this pattern with respect to the log_2_-transformed ratios (tumors/controls), and found a similar pattern (Figure 11). K-means clustering (Figure 11A–C) manifested four clusters, where cluster 1 (Figure 11D) and cluster 3 (Figure 11E) manifested the same interesting behavior as in the previously observed behavior; yet, when the control samples were included, the only significant difference observed was between grades I and IV (Figure 11E). However, in the case of cluster 4, tumor grade appeared to play a role in miRNA expression, in an ascending order from grade I to grade III, followed by tumors of grade IV (Figure 11F). Significant differences were manifested between tumor grades, as presented in Figure 11F.

### 3.7. Functional Analysis of DE miRNAs

The next step included the examination of the commonly expressed miRNAs, as well as those miRNAs that manifested expression patterns in each of the above-mentioned tumor catogories, for their functional properties.

#### 3.7.1. Gene Ontology Enrichment Analysis of Commonly Expressed miRNAs

**Figure 12:**
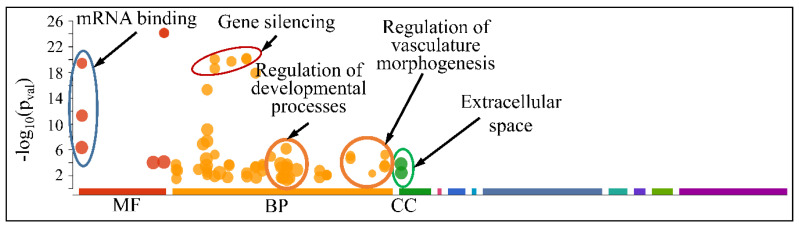
Functional annotation (GO enrichment analysis) of miRNAs found to be globally deregulated across all CNS tumor samples (Legend: MF: Molecular Function, BP: Biological Process, CC: Cellular Component).

The globally co-DE miRNAs (i.e., up- or down-regulated across all CNS tumors) were examined for their functional profiles, using Gene Ontology (GO) enrichment analysis. As expected, the co-DE miRNAs were also involved in mRNA binding (Molecular Function), gene silencing, regulation of developmental processes and vasculature morphogenesis (Biological Process) and were located in the extracellular space (Cellular Component) (Figure 12).

**Figure 13:**
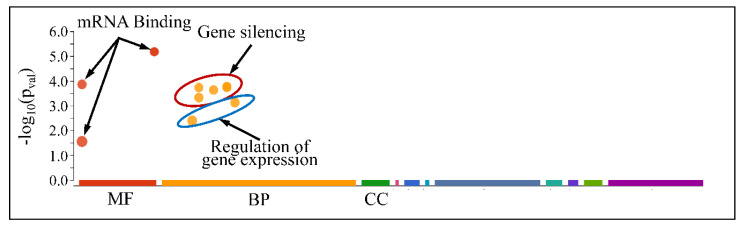
Functional annotation of miRNAs found globally up-regulated in all tumor samples (Legend: MF: Molecular Function, BP: Biological Process, CC: Cellular Component).

**Figure 14:**
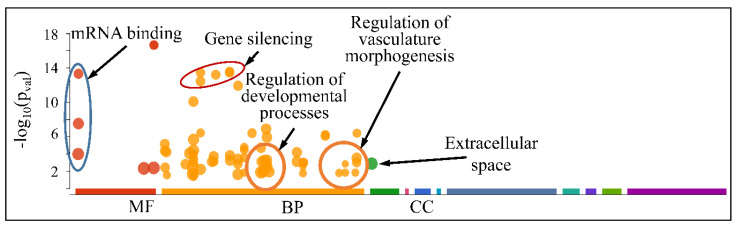
Functional annotation of miRNAs found globally down-regulated in all tumor samples (Legend: MF: Molecular Function, BP: Biological Process, CC: Cellular Component).

However, when separating the DE miRNAs into those being globally up-regulated and down-regulated, we found that the first were annotated in less functions, which included mRNA binding (Molecular Function) and gene silencing and the regulation of gene expression (Biological Process) (Figure 13); meanwhile, the latter further participate in the regulation of developmental processes and vasculature morphogenesis (Biological Process) (Figure 14). It is noteworthy that it appeared that there was a distinct separation between the co-up- and down-regulated miRNAs with respect to their annotated functions, signifying that down-regulated miRNAs affect more developmental properties and the regulation of cell proliferation.

#### 3.7.2. The Special Case of Down-Regulated miRNAs across All CNS Tumor Samples

**Figure 15:**
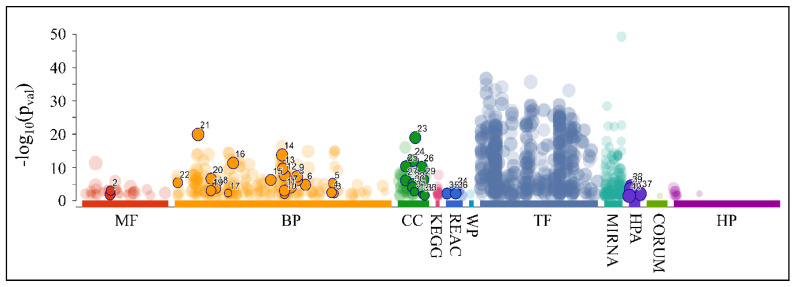
Functional annotation of the predicted mRNA targets for the co-downregulated miRNAs: MIR149, MIR214, MIR574, MIR595 and MIR765 (Legend: MF: Molecular Function, BP: Biological Process, CC: Cellular Component, KEGG: KEGG pathway database, REAC: Reactome pathway database, WP: WikiPathways, TF: Transcription Factor Binding Motifs, MIRNA: MiRNA Targets, HPA: The Human Protein Atlas, CORUM: The Comprehensive Resource of Mammalian Protein Complexes, HP: Human Phenotype Ontology).

As aforementioned, our search for common miRNAs revealed five miRNAs (MIR149, MIR214, MIR574, MIR595 and MIR765) that were down-regulated across all CNS tumor samples (100% of all cases). Target prediction analysis showed that these miRNAs had 2893 unique mRNA targets. When examining the functional annotation of their mRNA targets, we found that they participated in functions such as neuronal morphogenesis (nervous system development, generation of neurons, neuron projection development and neuron projection morphogenesis, among others), axon formation, synaptic function, as well as developmental processes (Figure 15 and Table 4).

#### 3.7.3. Functional Analysis of the DE miRNAs Manifested Expression Patterns

**Figure 16:**
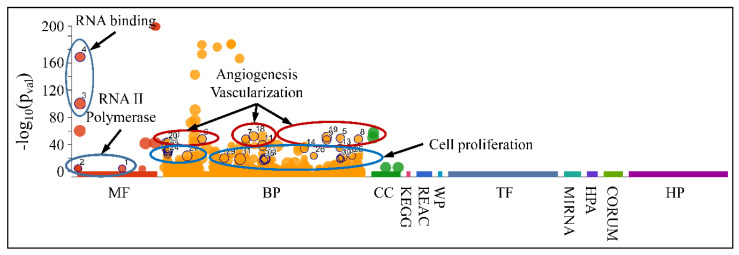
Functional annotation of miRNAs found to manifest an expressional pattern with respect to the tumor grade. (Legend: MF: Molecular Function, BP: Biological Process, CC: Cellular Component, KEGG: KEGG pathway database, REAC: Reactome pathway database, WP: WikiPathways, TF: Transcription Factor Binding Motifs, MIRNA: MiRNA Targets, HPA: The Human Protein Atlas, CORUM: The Comprehensive Resource of Mammalian Protein Complexes, HP: Human Phenotype Ontology).

Accordingly, we searched for the functional properties of the DE miRNAs with respect to the ascending patterns observed either for diagnosis (Figure 9) or tumor grade (Figure 10 and Figure 11). The DE miRNAs that participated in cluster 4 (Figure 9) did not manifest any significant functional annotations. On the contrary, the miRNAs that manifested an expression pattern with respect to the tumor grade (Figure 10 and Figure 11) were found to participate in functions such as angiogenesis, blood vessel developmental processes and vascularization, as well as cell proliferation (Figure 16). This was an interesting finding, since it appeared that miRNAs participating in tumor grading were related to angiogenesis, a significant characteristic of tumor growth.

### 3.8. ROC Analysis of Globally Down-Regulated miRNAs

**Figure 17:**
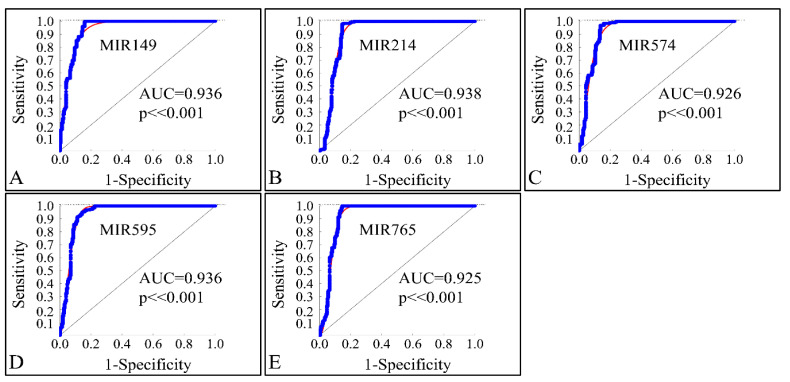
ROC analysis of MIR149 (**A**), MIR214 (**B**), MIR574 (**C**), MIR595 (**D**) and MIR765 (**E**). The miRNAs could successfully discriminate the controls and neoplasmatic CNS tumor samples with an area under the curve (AUC) ≥ 0.925 (*p* < 0.001), corroborating their use as diagnostic markers (Legend: AUC: Area under the Curve).

In order to investigate the diagnostic ability of the globally down-regulated miRNAs, we also performed a receiver operating characteristic curve (ROC) analysis with respect to the control and neoplasmatic samples. Confirming our previous observations, the five co-downregulated miRNAs (MIR149, MIR214, MIR574, MIR595 and MIR765) could successfully discriminate the controls and CNS tumor samples with an area under the curve (AUC) ≥ 0.925 (*p* < 0.001), corroborating their use as diagnostic markers (Figure 17A–E).

## 4. Discussion

MicroRNAs are considered as key modulating molecules in cellular epigenetic processes. In addition, they offer insight to many processes in normal and tumor cells. However, the study of miRNAs often involves hybridization-based microarray technologies, a high-throughput technology, that may generate a large opportunity for errors when used for testing their expression. Due to such limitations, all experiments must be regulated and controlled to reduce the chances of error regarding the produced data. In addition, other technologies must be implemented as a way to confirm the results of the microarrays.

In the present approach we used high throughput expression data and processed them to find common expression patterns between different childhood CNS tumors. There are numerous works concerning the role of miRNAs in CNS tumors, such as childhood embryonal tumors [1,18,40,41,42,43,44,45,46], astrocytoma [24,47,48], glioblastoma [49,50,51], ependymoma [52] and others. Yet, all the previous studies concerned the role of miRNAs in specific tumor types.

We followed a different approach, in which we introduced a novel concept of examining the role of miRNAs, simultaneously in the majority of the different CNS tumor types. To the best of our knowledge, there are no previous works similar to this approach. Although this type of approach could appear bold, it has several advantages. The identification of global biomarkers for CNS tumors is very useful, since CNS tumors manifest a challenge both due to their anatomic position as well as their severity. On the other hand, therapy constitutes a real peril for patients, since even in the cases of benign neoplasms both surgery and chemotherapy could prove dangerous for the patient. Thus, our skepsis leans towards the identification of biomarkers that could be used in all CNS tumor cases. Is such an approach possible? It could prove to be a tedious task. Yet, our reasoning is based on the hypothesis that tumors, irrespective of their type, follow a common machinery (at least to an extent), which is still unidentifiable. No matter how different the tumor is, which varies even from one patient to another, there are common signatures that lead oncogenesis, but most importantly tumor ontogenesis. Therefore, the identification of such biomarkers could be facilitated by similar approaches, such as the one presented here.

We found previously uncharacterized miRNA genes in pediatric CNS tumors, including MIR149, MIR214, MIR574 and MIR765, apart from one study that reported MIR595 upregulation in glioblastoma compared to control cells, in vitro [53]. Other miRNAs were also previously reported to regulate epithelial-to-mesenchymal transition (EMT) [54,55] and participate in CNS tumors, such as MIR34A. The overexpression of miR-34a was previously observed in pediatric ependymomas [56], pediatric pilocytic astrocytoma [17] and in pediatric low- and high-grade astrocytoma [57], suggesting that it plays a global oncogenic role in pediatric brain malignancies. miR-34 has been reported to be dysregulated in various human cancers and regarded as a tumor suppressive microRNA because of its synergistic effect with the well-known tumor suppressor p53 [58]. However, in this study MIR34A was not within the top co-DE miRNAs.

Before further discussing the individual miRNAs with respect to the literature, we need to note an interesting remark. Our sampling consisted of a large variety of CNS tissues, where some included fetal and neonatal samples. It is certain that the developing brain has a dynamic transcriptomic profile, which changes both spatially (with respect to brain location) as well as temporally [21]. Thus, it is possible that an analysis comparing tumor samples to normal brain tissue entails the problem of discovering different genes due to the tissue’s developmental stages and not due to the differences in pathology. First of all, it is important to highlight that in the case of CNS tumors one of the greatest problems is the obtainment of control samples, since biopsies of normal CNS tissue are extremely rare and difficult. Therefore, all available normal CNS samples are not to be unthoughtfully disregarded. Therefore, our first observation is that such a possibility cannot be ruled out. In order to completely remove this problematic, it is imperative to match samples (patients and controls) by age or use as controls adjacent (to the tumor) normal tissue. However, both approaches have their disadvantages. The first is that using only specific age groups restricts the number of available samples as well as the global temporal dynamics of gene regulation. On the other hand, using adjacent tissue that is considered to be normal has the disadvantage of including micro-environmental effects of the tumor on the nearby tissue [59,60,61]. A global approach has the disadvantage of identifying genes similar to both tissues with respect to their developmental machinery, yet this could also be an advantage. Another concept that we have to bear in mind is that tumors utilize a large part of the developmental machinery for their ontogenesis (and probably oncogenesis), which attributes to both normal developmental tissues and CNS tumors a common transcription profile [62,63,64,65,66]. Let us suppose that differentially expressed genes are found due to the difference in age (for example fetal/neonatal vs. child) and not the pathological state. The presence of developmental genes as differentially expressed indicates first of all that in tumors, developmental processes are active and most importantly they are different from one type of tissue to the other and secondly, that the tumor utilizes similar growth mechanisms to normal brain development [64,67]. Further on, in our study we have found that globally up- or down-regulated miRNAs manifested developmental-like functions, which confirmed the aforementioned reasoning. In addition, the identified developmental processes were attributed probably to the globally down-regulated miRNAs, which indicated that tumor tissues were utilizing the developmental machinery. Finally, there is a diversity in the use of control samples throughout the literature, where several studies have used age-matched [22] samples and others did not [19,20,24].

Interestingly, the majority of the co-DE miRNAs were found to be up-regulated (*n* = 261), and less down-regulated (*n* = 145). Yet, the co-down-regulated miRNAs manifested their DE profile in more tumor samples compared to the up-regulated ones. For example, five miRNAs were found to be down-regulated simultaneously across all CNS tumors, while no miRNA was found to be up-regulated in all the tumor samples. It is noteworthy that the down-regulated miRNAs participated in more functions, including the regulation of developmental processes, while the up-regulated miRNAs participated in many less functions. Yet, the up-regulated miRNAs are of greater importance with respect to therapy, since the over-expressed miRNAs can be inhibited using oligonucleotides that are perfectly complementary to their mature miRNA targets, whereas the transfection of miRNA mimics (chemically synthesized double-stranded RNA molecules) to imitate mature miRNA duplexes, a more difficult task. The difficulty lies in that it is unclear whether the transfected miRNAs behave similarly to endogenous miRNAs.

Although many of the up-regulated miRNAs that we found are not known to participate in CNS tumors, MIR183 has been previously reported to be up-regulated in glioblastoma [68] and glioma [69,70,71]. In addition, there are two contradictory reports regarding MIR433. One study reported that MIR433 down-regulation is connected to tumor suppression [72], which was in agreement with our study, whereas another mentions that MIR433 up-regulation has tumor suppressive effects [73]. Regarding MIR519D we found only one report, stating that this miRNA is down-regulated in CNS tumors [74], conferring tumor suppressive properties; in contrast, we found that MRI519D is overexpressed in the majority of the CNS tumors. At the same time, MIR518B was up-regulated and reported to manifest tumor suppressive properties in glioblastoma [75,76]. Interestingly, MIR367 was recently found to function as tumor promoting miRNA, since its inhibition attenuates tumor aggressiveness and proliferation in embryonal tumors [77,78,79]. On the other hand, MIR613 was reported to act as a tumor suppressor, inhibiting glioma progression [80], while we found it up-regulated in the majority of the CNS tumors. Similarly, MIR216A has been reported to manifest tumor suppressing properties in glioma cells, yet it is also found that its role has been contradictory, as it participates either as tumor suppressor or oncogene depending on the tumor type [81]. In our study, MIR216A was up-regulated in the majority of the examined CNS tumors, suggesting that it functions as an oncogene. A contradictory finding concerned MIR599, which was found to be upregulated and probably act as an oncogene. Yet, MIR599 was reported in two studies to act as a tumor suppressor in glioma tumors, as it was found to be down-regulated [82,83]. Finally, MIR577 [84,85,86] and MIR429 [87,88] were previously reported to act as tumor suppressors in gliomas, while we have found them to function as oncogenes in the global setting of CNS tumors.

The present approach is reported previously in the literature. To the best of our knowledge, this is the first time such a reasoning is presented in the literature. In a previous work we have performed a similar approach for urinary bladder cancer, where we have reported that a single gene (CDC20) manifested a common profile among different subtypes of urothelial bladder cancer [89]. In the meantime, CDC20 was confirmed to be a molecule of interest for prognosis and therapy [90,91,92]. Thus, the present approach could also prove useful for the detection of therapeutic strategies. One drawback to the present approach is that several of the identified miRNAs are still unknown for their role in CNS tumors and, therefore, a series of functional investigations are required not limited to the validation of the expressional profile, but extended to the actual verification of miRNA targets.

In summary, the current study provides significant insights in the growing role of miRNA signatures in pediatric CNS neoplasms of different type, such as medulloblastomas, ATRTs, astrocytomas, ependymomas, glioblastomas and others. In general, we found good evidence that miRNAs manifest global patterns of co-deregulated expression across all CNS tumor types. Collectively, our findings highlight miRNAs that could be used as novel molecular biomarkers with a promising potential in pediatric CNS malignancies.

## 5. Conclusions

The present study proposed a novel approach to investigate the miRNA-related mechanisms across different types of pediatric CNS tumors. Interestingly, we found miRNAs that were globally down- and up-regulated in all CNS tumor samples. Our approach could be useful in the discovery of novel therapeutic markers for CNS tumors, yet further research is required in order to confirm miRNAs’ functions.

## Figures and Tables

**Table 1:** Summary of the microarray experiments (data series) used in the present study (Legend: GCTs: Intracranial pediatric germ cell tumors).

Series	Platform	Diagnosis	Sample Number	Publication
GSE19347	GPL8227	GCTs-Germinoma	6	Wang et al. (2010) [19]
GSE19347	GPL8227	GCTs-Teratoma	3	Wang et al. (2010) [19]
GSE19347	GPL8227	GCTs-Yoc sac tumor	3	Wang et al. (2010) [19]
GSE34016	GPL8786	Control (Neural progenitor cells)	6	N/A
GSE42657	GPL8179	Pilocytic Astrocytoma	15	Jones et al. (2015) [20]
GSE42657	GPL8179	Papillary Neuroglial Tumor	1	Jones et al. (2015) [20]
GSE42657	GPL8179	Diffuse Astrocytoma	3	Jones et al. (2015) [20]
GSE42657	GPL8179	Anaplastic Astrocytoma	2	Jones et al. (2015) [20]
GSE42657	GPL8179	Glioblastoma	5	Jones et al. (2015) [20]
GSE42657	GPL8179	Ependymoma	14	Jones et al. (2015) [20]
GSE42657	GPL8179	Medulloblastoma	9	Jones et al. (2015) [20]
GSE42657	GPL8179	Atypical teratoid rhabdoid tumor (ATRT)	5	Jones et al. (2015) [20]
GSE42657	GPL8179	Choroid Plexus Papilloma	4	Jones et al. (2015) [20]
GSE42657	GPL8179	Controls	7	Jones et al. (2015) [20]
GSE45126	GPL16783	Controls (Mixture of all RNA samples)	98	Moreau et al. (2013) [21]
GSE45126	GPL16783	Controls (Fetal brain)	98	Moreau et al. (2013) [21]
GSE62367	GPL16384	Ocular Medulloepithelioma	5	Edward et al. (2015) [22]
GSE62367	GPL16384	Controls	8	Edward et al. (2015) [22]
GSE63319	GPL16384	Glioblastoma	11	N/A
GSE63319	GPL16384	Anaplastic Astrocytoma	3	N/A
GSE63319	GPL16384	Controls	4	N/A
GSE66968	GPL8227	Medulloblastoma	29	N/A
GSE84747	GPL21572	Retinoblastoma	12	Castro-Magdonel et al. (2017) [23]
GSE135189	GPL20906	Pilocytic Astrocytoma	16	Darrigo et al. (2019) [24]
GSE135189	GPL20906	Ocular Medulloepithelioma	1	Darrigo et al. (2019) [24]
GSE135189	GPL20906	Controls	11	Darrigo et al. (2019) [24]
In-house	miRLink (https://appliedmicroarrays.com/, Last Accessed on 5 September 2020)	Pilocytic Astrocytoma	19	Braoudaki et al. (2016) [25]
In-house	miRLink (https://appliedmicroarrays.com/, Last Accessed on 5 September 2020)	Ependymoma	7	Braoudaki et al. (2016) [25]
In-house	miRLink (https://appliedmicroarrays.com/, Last Accessed on 5 September 2020)	Medulloblastoma	15	Braoudaki et al. (2014) [18]
In-house	miRLink (https://appliedmicroarrays.com/, Last Accessed on 5 September 2020)	ATRT	4	Braoudaki et al. (2014) [18]
In-house	miRLink (https://appliedmicroarrays.com/, Last Accessed on 5 September 2020)	Cortical Dysplasia	2	Braoudaki et al. (2014) [18]
In-house	miRLink (https://appliedmicroarrays.com/, Last Accessed on 5 September 2020)	Controls	14	Braoudaki et al. (2014, 2016) [18,25]

**Table 2:** The sample cohort that was used in the present study.

Primary Nominal Variables	Secondary Nominal Variables	*N*	Descriptive Variables	Age (Years) ^¥^	Gest Age (Years) ^α^
Total Population		439	Mean ± SD	4.20 ± 5.24	4.84 ± 5.31
			Median (range)	1.73 (0.00–27.00)	2.47 (0.19–27.74)
Gender ^1^	FEMALES	97	Mean ± SD	3.99 ± 4.76	4.60 ± 4.88
			Median	2.00 (0.00–16.00)	2.74 (0.27–16.74)
	MALES	165	Mean ± SD	5.55 ± 6.32	6.19 ± 6.41
			Median	3.00 (0.00–27.00)	3.74 (0.19–27.74)
	FETUS	6	Mean ± SD	0.00 ± 0.00	0.23 ± 0.00
			Median	0.00 (0.00–0.00)	0.23 (0.23–0.23)
	Not Available	171	NaN	NaN	NaN
Sampling ^2^	VIVUS	181	Mean ± SD	7.86 ± 5.62	8.60 ± 5.62
			Median	7.00 (0.03–27.00)	7.74 (0.77–27.74)
	POST-MORTEM	200	Mean ± SD	0.89 ± 0.84	1.46 ± 1.02
			Median	0.73 (0.00–1.73)	1.45 (0.19–2.47)
First Diagnosis ^3^	NEOPLASM	195	Mean ± SD	7.29 ± 4.93	8.03 ± 4.93
			Median	7.00 (0.03–19.00)	7.74 (0.77–19.74)
	CONTROL	244	Mean ± SD	1.69 ± 4.01	2.26 ± 4.07
			Median	1.73 (0.00–27.00)	2.47 (0.19–27.74)
Second Diagnosis ^4^	MALIGNANCY	154	Mean ± SD	7.55 ± 5.05	8.29 ± 5.05
			Median	7.00 (0.03–19.00)	7.74 (0.77–19.74)
	BENIGN	41	Mean ± SD	6.35 ± 4.41	7.09 ± 4.41
			Median	6.00 (0.83–16.00)	6.74 (1.57–16.74)
	CONTROL	244	Mean ± SD	1.69 ± 4.01	2.26 ± 4.07
			Median	1.73 (0.00–27.00)	2.47 (0.19–27.74)
Third Diagnosis ^5^	MEDULLOBLASTOMA	53	Mean ± SD	6.38 ± 4.06	7.12 ± 4.06
			Median	6.00 (0.50–16.06)	6.74 (1.24–16.80)
	ASTROCYTOMA	58	Mean ± SD	8.08 ± 5.14	8.82 ± 5.14
			Median	7.00 (0.92–19.00)	7.74 (1.66–19.74)
	EPENDYMOMA	21	Mean ± SD	5.19 ± 4.34	5.93 ± 4.34
			Median	4.00 (1.00–16.01)	4.74 (1.74–16.75)
	ATRT	9	Mean ± SD	1.59 ± 2.37	2.33 ± 2.37
			Median	0.75 (0.03–7.61)	1.49 (0.77–8.35)
	CONTROL	244	Mean ± SD	1.69 ± 4.01	2.26 ± 4.07
			Median	1.73 (0.00–27.00)	2.47 (0.19–27.74)
	CORTICAL DYSPLASIA	2	Mean ± SD	10.78 ± 3.87	11.52 ± 3.87
			Median	10.78 (8.04–13.52)	11.52 (8.78–14.25)
	GLIOBLASTOMA	16	Mean ± SD	12.73 ± 2.29	13.47 ± 2.29
			Median	12.75 (10.40–15.90)	13.49 (11.14–16.64)
	GERMINOMA	6	Mean ± SD	4.86 ± 5.13	5.60 ± 5.13
			Median	4.30 (0.03–10.25)	5.04 (0.77–10.99)
	TERATOMA	3	Mean ± SD	10.93 ± 3.41	11.67 ± 3.41
			Median	10.60 (7.70–14.50)	11.34 (8.44–15.24)
	YOC SAC TUMOR	3	Mean ± SD	14.00 ± 0.00	14.74 ± 0.00
			Median	14.00 (14.00–14.00)	14.74 (14.74–14.74)
	GLIONEURONAL	1	Mean ± SD	11.31 ± 3.75	12.05 ± 3.75
			Median	12.00 (4.00–18.00)	12.74 (4.74–18.74)
	PAPILLOMA	4	Mean ± SD	1.61 ± 1.21	2.35 ± 1.21
			Median	1.00 (0.83–3.00)	1.74 (1.57–3.74)
	OCULAR TUMOR ^5a^	6	Mean ± SD	NaN	NaN
			Median	NaN	NaN
	RETINOBLASTOMA ^5b^	12	Mean ± SD	NaN	NaN
			Median	NaN	NaN
Grade ^6^	I	53	Mean ± SD	7.53 ± 4.96	8.27 ± 4.96
			Median	6.69 (0.83–19.00)	7.43 (1.57–19.74)
	II	18	Mean ± SD	6.85 ± 5.26	7.59 ± 5.26
			Median	4.47 (0.26–16.01)	5.21 (1.00–16.75)
	III	12	Mean ± SD	4.19 ± 3.88	4.93 ± 3.88
			Median	2.55 (1.00–15.00)	3.29 (1.74–15.74)
	IV	92	Mean ± SD	7.64 ± 4.90	8.38 ± 4.90
			Median	7.82 (0.03–18.00)	8.56 (0.77–18.74)
	CONTROL	244	Mean ± SD	1.69 ± 4.01	2.26 ± 4.07
			Median	1.73 (0.00–27.00)	2.47 (0.19–27.74)
	Not Available	20	Mean ± SD	NaN	NaN
			Median	NaN	NaN
Developmental Status ^7^	CHILD	125	Mean ± SD	6.13 ± 3.29	6.87 ± 3.29
			Median	6.00 (0.47–12.00)	6.74 (1.21–12.74)
	INFANT	37	Mean ± SD	0.81 ± 0.65	1.55 ± 0.65
			Median	0.74 (0.16–2.02)	1.48 (0.90–2.76)
	NEONATE	4	Mean ± SD	0.02 ± 0.01	0.76 ± 0.01
			Median	0.02 (0.01–0.03)	0.76 (0.75–0.77)
	ADOLESCENT	43	Mean ± SD	13.38 ± 3.24	14.12 ± 3.24
			Median	14.00 (1.90–18.00)	14.74 (2.64–18.74)
	ADULT	6	Mean ± SD	0.01 ± 0.08	0.35 ± 0.13
			Median	0.00 (0.00–0.71)	0.35 (0.19–1.45)
	FETUS	81	Mean ± SD	23.33 ± 3.14	24.07 ± 3.14
			Median	23.50 (19.00–27.00)	24.24 (19.74–27.74)
	ALL STAGES	100	Mean ± SD	1.73 ± 0.00	2.45 ± 0.21
			Median	1.73 (1.73–1.73)	2.47 (0.38–2.47)

(Legend: ^¥^ age (years), ^α^ Gest. Age: gestational age, which is calculated as the age in years plus a mean gestational period of nine months, or the gestational period provided if the sample was obtained from a fetus. ^1^ Gender included fetuses if the age of the fetus was provided; ^2^ describes the status of the patient at the time of biopsy, i.e., if biopsy was taken from a live subject or post-mortem; ^3^ diagnosis separated into only two categories, i.e., neoplasm or control; ^4^ diagnosis separated into only three categories, i.e., malignant, benign and controls; ^5^ actual diagnosis (does not include subtypes). ^5a and 5b^ Although both tumors originate in the eye, we have included them due to their ectodermal origin; ^6^ tumor grade; ^7^ fetus: during gestational period; neonate: 28 days old, infant: from 28 days old to 1 year old; child: 1–12 years old; adol: adolescent 12–17 years old; adult: >18 years old; all stages: concerns the samples provided by dataset GSE45126, in which a mixture of all RNAs was used as a control cohort; NaN, no values available).

**Table 3:** Common up- and down-regulated miRNAs across all CNS tumors. K-means clusters were also analyzed for the presence of co-DE miRNAs in all samples. MiRNAs were considered to manifest a common pattern of expression if they were globally up-regulated in at least >75% of all samples or down-regulated in at least >90% of all samples. All miRNAs were sorted with respect to their cluster presence.

Inv.	miRNA	Pattern	*f*	*f* (%)	K-Means Cluster	Mean Expression
1	MIR376B	Up-regulated	165	84.61	1	0.722
2	MIR372	Up-regulated	148	75.90	1	0.624
3	MIR149	Down-regulated	195	100.00	2	−5.491
4	MIR214	Down-regulated	195	100.00	2	−4.742
5	MIR574	Down-regulated	195	100.00	2	−4.975
6	MIR595	Down-regulated	195	100.00	2	−4.083
7	MIR765	Down-regulated	195	100.00	2	−5.690
8	MIR92B	Down-regulated	182	93.33	2	−3.622
9	MIR939	Down-regulated	189	96.92	2	−3.225
10	MIR202	Down-regulated	190	97.43	2	−3.369
11	MIR921	Down-regulated	191	97.95	2	−3.527
12	MIR494	Down-regulated	193	98.97	2	−6.053
13	MIR665	Down-regulated	193	98.97	2	−4.835
14	MIR936	Down-regulated	193	98.97	2	−4.799
15	MIR575	Down-regulated	194	99.49	2	−3.731
16	MIR638	Down-regulated	194	99.49	2	−5.799
17	MIR933	Down-regulated	194	99.49	2	−4.271
18	MIR32	Down-regulated	176	90.25	3	−1.7540219
19	MIR557	Down-regulated	176	90.25	3	−1.2031172
20	MIR583	Down-regulated	177	90.77	3	−1.3094348
21	MIR675	Down-regulated	177	90.77	3	−1.8494352
22	MIR370	Down-regulated	178	91.28	3	−1.7722773
23	MIR760	Down-regulated	178	91.28	3	−1.4657573
24	MIR198	Down-regulated	179	91.80	3	−2.2433618
25	MIR210	Down-regulated	179	91.80	3	−2.3769263
26	MIR627	Down-regulated	179	91.80	3	−1.0966398
27	MIR650	Down-regulated	179	91.80	3	−2.0081156
28	MIR647	Down-regulated	180	92.31	3	−1.6223603
29	MIR632	Down-regulated	181	92.82	3	−1.5932408
30	MIR498	Down-regulated	183	93.85	3	−1.7145673
31	MIR608	Down-regulated	184	94.36	3	−2.4389162
32	MIR610	Down-regulated	184	94.36	3	−1.9065001
33	MIR564	Down-regulated	185	94.87	3	−2.1568429
34	MIR206	Down-regulated	186	95.38	3	−2.9338987
35	MIR671	Down-regulated	186	95.38	3	−1.6370801
36	MIR297	Down-regulated	188	96.41	3	−2.8914463
37	MIR637	Down-regulated	188	96.41	3	−3.1802687
38	MIR891A	Down-regulated	188	96.41	3	−2.5938886
39	MIR185	Down-regulated	193	98.97	3	−2.8633022
40	MIR183	Up-regulated	176	90.26	4	−1.7540219
41	MIR889	Up-regulated	176	90.26	4	−1.2031172
42	MIR520H	Up-regulated	177	90.77	4	−1.3094348
43	MIR563	Up-regulated	177	90.77	4	−1.8494352
44	MIR433	Up-regulated	178	91.28	4	−1.7722773
45	MIR519D	Up-regulated	178	91.28	4	−1.4657573
46	MIR891B	Up-regulated	179	91.79	4	−2.2433618
47	MIR631	Up-regulated	179	91.79	4	−2.3769263
48	MIR518B	Up-regulated	179	91.79	4	−1.0966398
49	MIR367	Up-regulated	179	91.79	4	−2.0081156
50	MIR613	Up-regulated	180	92.31	4	−1.6223603
51	MIR651	Up-regulated	181	92.82	4	−1.5932408
52	MIR216A	Up-regulated	183	93.85	4	−1.7145673
53	MIR374A	Up-regulated	184	94.36	4	−2.4389162
54	MIR599	Up-regulated	184	94.36	4	−1.9065001
55	MIR577	Up-regulated	185	94.87	4	−2.1568429
56	MIR190B	Up-regulated	186	95.38	4	−2.9338987
57	MIR429	Up-regulated	186	95.38	4	−1.6370801
58	MIR567	Up-regulated	188	96.41	4	−2.8914463
59	MIR618	Up-regulated	188	96.41	4	−3.1802687
60	MIR581	Up-regulated	188	96.41	4	−2.5938886
61	MIR183	Up-regulated	193	98.97	4	−2.8633022

**Table 4:** The respective functional annotation of the predicted mRNA targets for the co-downregulated miRNAs: MIR149, MIR214, MIR574, MIR595 and MIR765. The colors correspond to the annotation colors presented in Figure 14 (Legend: MF: Molecular Function, BP: Biological Process, CC: Cellular Component, KEGG: KEGG pathway database, REAC: Reactome pathway database, HPA: The Human Protein Atlas).

Source	Term Name	−log_10_(*p*)
GO:MF	SH3 domain binding	2.936
GO:MF	Ras GTPase binding	1.731
GO:BP	nervous system development	19.815
GO:BP	generation of neurons	13.643
GO:BP	neuron projection development	11.23
GO:BP	neuron projection morphogenesis	9.512
GO:BP	regulation of nervous system development	8.701
GO:BP	central nervous system development	6.965
GO:BP	cell morphogenesis involved in neuron differentiation	6.74
GO:BP	regulation of neuron projection development	6.543
GO:BP	axonogenesis	5.644
GO:BP	synaptic vesicle cycle	4.855
GO:BP	axon development	4.648
GO:BP	neurotransmitter transport	4.407
GO:BP	regulation of synaptic plasticity	2.796
GO:BP	neurotransmitter secretion	2.601
GO:BP	regulation of axonogenesis	2.352
GO:BP	neuron projection guidance	2.344
GO:BP	dendritic spine development	2.197
GO:BP	central nervous system neuron axonogenesis	2.195
GO:BP	synaptic vesicle exocytosis	2.078
GO:BP	central nervous system neuron differentiation	1.375
GO:BP	central nervous system projection neuron axonogenesis	1.345
GO:BP	camera-type eye morphogenesis	1.332
GO:CC	synapse	18.837
GO:CC	neuron projection	11.917
GO:CC	somatodendritic compartment	11.311
GO:CC	dendrite	10.215
GO:CC	dendritic tree	10.092
GO:CC	neuron spine	3.125
GO:CC	postsynaptic membrane	2.872
GO:CC	neuron projection terminus	2.794
GO:CC	main axon	2.58
KEGG	Pathways in cancer	7.647
KEGG	Axon guidance	4.293
KEGG	MicroRNAs in cancer	3.479
REAC	Neuronal System	3.262
REAC	Axon guidance	2.046
REAC	Nervous system development	2.046
REAC	Transmission across Chemical Synapses	1.421
REAC	Vesicle-mediated transport	1.391
HPA	cerebral cortex; neuropil [Approved, Medium]	4.339
HPA	cerebral cortex; neuropil [Approved, Low]	3.99
HPA	cerebellum; cells in granular layer [Approved, Medium]	3.958
HPA	cerebellum; cells in granular layer [Approved, Low]	3.439
HPA	hippocampus; neuronal cells [Approved Low]	1.66
HPA	cerebral cortex; neuropil [Approved, High]	1.507
HPA	cerebral cortex; neuronal cells [Approved, Low]	1.47

## Data Availability

Data are available from the corresponding author upon reasonable request.

## Supplementary Materials

The following are available online at https://www.mdpi.com/article/10.3390/cancers13123028/s1, Table S1: Quantile normalized microarray data.
